# Consent for Medical Treatment: What is ‘Reasonable’?

**DOI:** 10.1007/s10728-023-00466-8

**Published:** 2023-08-19

**Authors:** Abeezar Ismail Sarela

**Affiliations:** Department of Surgery, The Leeds Teaching Hospitals NHS Trust, Beckett Street, Leeds, UK

**Keywords:** Informed consent, Clinical negligence, Test of materiality, *Montgomery v Lanarkshire*, *Bolam*, General Medical Council

## Abstract

The General Medical Council (GMC) instructs doctors to act ‘reasonably’ in obtaining consent from patients. However, the GMC does not explain what it means to be reasonable: it is left to doctors to figure out the substance of this instruction. The GMC relies on the Supreme Court’s judgment in *Montgomery v Lanarkshire Health Board*; and it can be assumed that the judges’ idea of reasonability is adopted. The aim of this paper is to flesh out this idea of reasonability. This idea is commonly personified as the audience that has to be satisfied by the doctor’s justification for offering, or withholding, certain treatments and related information. In case law, this audience shifted from a reasonable doctor to a ‘reasonable person in the patient’s position’; and *Montgomery* expands the audience to include ‘particular’ patients, too. Senior judges have clarified that the reasonable person is a normative ideal, and not a sociological construct; but they do not set out the characteristics of this ideal. John Rawls has conceived the reasonable person-ideal as one that pursues fair terms of co-operation with other members of society. An alternative ideal can be inferred from the feminist ethic of care. However, the reasonable patient from *Montgomery* does not align with either theoretical ideal; but, instead, is an entirely rational being. Such a conception conflicts with both real-life constraints on rationality and the doctor’s duty to care for the patient, and it challenges the practice of medicine.

## Introduction

NHS Resolution (NHSR; formerly, the NHS’s Litigation Authority) reports that claims of clinical negligence in obtaining patients’ consent for medical treatment have cost £ 202 million over the past 5 years [1]: a 55% increase in expenditure as compared to the previous 5 years [2]. True costs, which include projected, outstanding liabilities, in addition to costs already incurred, are likely to be much higher [3]. Such expenditure represents a considerable financial burden for a cash-strapped NHS; and it presents an additional reason, over and above important medical ethical considerations, for clarifying doctors’ obligations in seeking and obtaining patients’ consent. For, it is a trite conclusion that, if doctors better understand and deliver their obligations, then there would be fewer allegations of negligence.

In the UK, doctors are regulated by the General Medical Council (GMC), and they rely on the GMC for guidance on how to discharge their professional obligations. The GMC issued new guidance on consent in November 2020 [4]. It has been argued that this guidance makes little substantive advance on the earlier (2008) guidance [5]. Yet, there is an important difference in emphasis: in the 2020 guidance, the word ‘reasonable’ appears 13 times; whereas, in the 2008 guidance it appeared only twice. Moreover, in the 2008 guidance, the advice to be ‘reasonable’ was limited to the contexts of assessing capacity and for best interests decision-making (paras 19, 25) [6]; whereas, the 2020 guidance clearly extends the obligation to be ‘reasonable’ to all aspects of consent (para 88) [4]. For instances, doctors are now advised to ‘give them (patients) all reasonable help and support to make a decision’ (para 82) and to inform patients about ‘reasonable alternatives’ (para 7) to any proposed treatment [4]. At the same time, the GMC makes it clear that there are limits on informational exchange because ‘It wouldn’t be reasonable to share every possible risk of harm, potential complication or side effect’ (para 22) [4]. Thus, being ‘reasonable’ now occupies a central role in the doctor’s obligation to obtain patients’ consent for treatment.

What is a doctor required to do in order to be ‘reasonable’? The GMC does not explicate the term. Although, it implies that that if the doctor has been reasonable then ‘then you will be able to explain and justify your decisions and actions’ (pg. 5) [4]. In other words, a doctor’s decisions and actions would be considered to be reasonable if she or he was able to provide justification for these decisions and actions. This inference chimes with John Gardner’s proposition that a reasonable decision or action is a justifiable decision or action [7]. He explains that justification is the provision of reasons that stand undefeated by conflicting reasons. Although, given the plurality of viewpoints in a diverse society, there may be disputes about whether certain factors count as reasons, at all; or whether certain reasons stand undefeated by conflicting reasons. A decision or action that seems justified, and hence reasonable, to some people may appear unjustified and unreasonable to others. As such, in order to anchor reasonableness to justification, it is necessary to identify a reference-point or standard of justification upon which all relevant people, regardless of their differing viewpoints, can agree.

The GMC does not elaborate on the standard of justification for a doctor’s decision or action to be regarded as reasonable. It is left to the doctor to figure out what it means to be reasonable. The GMC’s strategy is not surprising. In setting out its guidance on consent, the GMC relies on case law; and, as pointed out by Gardner, judges, too, have used the GMC’s ploy. He discusses that reliance upon reasonableness, without specification, ‘mitigates the awesome responsibility, for judges, of having to set legal standards that are fit for re-use in future cases’ (pg. 571) [8]. Now, judges are able to ‘pass the buck’ of responsibility in litigation to fact-finders^1^ to determine extra-legal standards that can be used flexibly on a case-by-case basis (pg. 571) [8]. As Gardner has explained, it would be impossible for lawmakers to set out a compendium of rules that could cover every conceivable situation; and, so, the retreat to reasonableness ‘is actually one of law’s clever devices to reopen a bit of space’ for contextual reasoning that could not possibly be covered otherwise (pg. 299) [7]. Likewise, the GMC would seem to have ‘passed the buck’ to doctors.

Whilst the GMC’s tactic is coherent with the law, it leaves doctors with the metaphorical ‘buck’: a position for which they are not specially prepared by professional training. The aim of the present paper is to equip doctors, and those who scrutinize doctors’ actions and decisions, to better understand the idea of reasonableness and the associated standard of justification in the context of consent for medical treatment. In short, in Gardner’s words, the aim is to ‘lend some substance to the reasonableness standard’ (pg. 288) [7]. The paper is arranged in the following sections. Section II discusses that the identification of the standard for justification reduces, ultimately, to the identification of the relevant audience—the constituency—to whom justification has to be provided. In case law, this justificatory constituency has shifted from the reasonable doctor to the reasonable person in the position of the patient, and it has expanded to include notions of both objectivity and subjectivity. Section III debates the alternative conceptions of a reasonable person—a sociologically-derived ‘ordinary’ person versus a normative ‘ideal’—that a doctor might adopt, and it highlights that senior judges have clearly endorsed the latter. Section IV sets out the influential theory of John Rawls on the ideal of a reasonable person. It also discusses the feminist challenge to Rawls and considers the alternative ideal that might emerge from the ethic of care that is foundational to the medical profession. Section V examines case law in the frameworks of the Rawlsian and the ethic of care ideals of a reasonable person, and it argues the judgment of the Supreme Court in the landmark case of *Montgomery v Lanarkshire Health Board* [9] does not employ either of these ideals. Section VI concludes this paper by highlighting the tensions in the substance of the reasonableness standard and the associated challenge for doctors.

## The Justificatory Constituency

Gardner discusses that the standard of being reasonable is commonly personified as a ‘reasonable person’. This person is, now, the audience, or constituency, who has to be satisfied with the justification for any decision or action [10]. As such, when contemplating any decision or action, the agent of reasonableness (the one who is required to behave reasonably) is ‘invited to think about a complete (albeit imaginary) person such as the reasonable person’(pg. 4) [10]; and to then consider whether the proposed action or decision could be justified to this reasonable person. Stated differently, the agent of reasonableness has to consider whether her reasons for any decision or action would be acceptable to this imaginary reasonable person as undefeated reasons; because, otherwise, the decision or action would not be regarded as reasonable. The agent has to endow this imaginary reasonable person with ‘a personality, a character and a temperament’, and to think about that person’s ‘established patterns of action, and dispositions towards action, and not just each of his or her actions in isolation’ (pg. 4) [10].

## The Shift in Constituency

As per the reasoning in the case of *Bolam v Friern Hospital* [11], later approved by the House of Lords in *Whitehouse v Jordan* [12], a doctor is required to conform to the ‘the standard of the ordinary skilled man exercising and professing to have that special skill’ (pg. 586) [11]. It follows that a doctor was required to be able to justify her decisions and actions to ‘the ordinary skilled man’ in her profession. As explained by Lord Scarman, a doctor was obliged to apply the standard of being ‘reasonable in the sense that a responsible body of medical opinion would have accepted it as proper’ (pg. 638 F) [13]. Thus, the *Bolam* paradigm obliges the doctor to imagine an ‘ordinary’ and ‘responsible’ group of her peers—the constituency to whom she is obliged to provide justification—and to act in a way that she or he would be able to justify to this constituency.

In approving the *Bolam* dicta, Lord Edmund-Davies held that the same justificatory constituency—the doctor’s peers—was engaged for all aspects of the doctor’s duty (pg. 258D) [12]. However, later, in *Sidaway v Bethlem Royal Hospital* [14], Lord Scarman rejected this constituency for the justification of a doctor’s duty to provide ‘advice’: a term that Lord Scarman used as a cognate for the doctor’s obligation to seek and obtain the patient’s consent, prior to embarking upon treatment. Instead, by reference to the USA case of *Canterbury v Spence* [15], he asserted that a doctor was obliged to advise the patient about all material risks of the proposed treatment, and that test of materiality asked whether:

*[A] reasonable person*,^2^ in what the physician knows or should know to be the patient’s position, would be likely to attach significance to the risk or cluster of risks in deciding whether or not to forego the proposed therapy’ (pg. 887D) [14].

Thus, Lord Scarman shifted the justificatory constituency from ‘a responsible body of medical opinion’ to a ‘reasonable person’.^3^ Furthermore, the constituency is not simply any reasonable person, but a reasonable person in the ‘patient’s position’. Now, when seeking a patient’s consent, a doctor is obliged to imagine, first, a reasonable person and, then, to place this imaginary person in the patient’s actual ‘position’; and, now, to be able to provide justification to this multi-faceted person.

## Expansion of the Constituency

In *Montgomery* [9], the Supreme Court resoundingly affirmed Lord Scarman’s approach. Although, Lord Kerr and Lord Reed JJSC introduced a ‘refinement’ (para 87) [9] by adopting the test of materiality from the judgment of the High Court of Australia in *Rogers v Whittaker* [16], rather than employing the test of materiality that had been articulated by Lord Scarman (adopted, in turn, from *Canterbury*). As per the *Rogers* test:

[A] risk is material if, in the circumstances of the particular case, a reasonable person in the patient’s position, if warned of the risk, would be likely to attach significance to it or if the medical practitioner is or should reasonably be aware that the particular patient, if warned of the risk, would be likely to attach significance to it (para 87) [9].

The *Rogers* test of materiality is explicated by Gummow J. in the Australian case of *Rosenberg v Percival* [17]; which, too, is cited approvingly in *Montgomery* (para 73) [9]. Gummow J. distinguished two limbs—objective and subjective—of the *Rogers* test (para 75) [17]. He explained that the objective limb was the first part of the *Rogers* test, which focusses on a reasonable patient in the patient’s position. The subjective limb is the second part, which deals with the ‘particular’ patient. Gummow J. highlighted that the subjective limb allows ‘that the particular patient may not be a “reasonable” one; he or she may have a number of “unreasonable” fears or concerns’ (para 79) [17]. Similarly, Callinan J. described the *Rogers* test as ‘a universal test for an hypothetical reasonable person in the patient’s position, and a test to be applied to the particular patient, even if, perhaps, she or he is an unreasonable one’ (para 210) [17]. Now, the doctor is required to provide justification to not only a reasonable person in the patient’s position but also to an unreasonable patient. It is this expansion of the justificatory audience to include the particular, ‘unreasonable’ patient that distinguishes the *Rogers* test from the *Canterbury* test; and this expansion represents the Supreme Court’s ‘refinement’ of Lord Scarman’s approach.

### Two Aspects of the Constituency: Objective and Subjective

The objective-subjective distinction in the courts’ reasonableness standard has been the subject of some academic discussion. A completely objective standard would assess decisions and actions ‘solely from the vantage of a reasonable person rather than from the actual thoughts and feelings of the actor’, that is, the individual patient (pg. 84) [18]. On the other hand, a completely subjective standard ‘includes all the individual characteristics of an actor and then evaluates whether the ignorance is reasonable for them’ (pg. 84) [18]. The dilemma, then, is to agree on the standard, that is, the conception of a reasonable patient, to whom justification has to be supplied. A completely objective standard risks depersonification of the individual patient by shoehorning her into the preconceived notion of a reasonable person; because, reasonable persons ‘are impersonal in that they do not bend to the varying personal characteristics of those who are judged by them’ (pg. 587) [8]. In contrast, unreasonable persons can have idiosyncratic and entirely unpredictable demands; and, a subjective standard, which allows unrestrained demands, would not supply a generalizable reference-point.

### The Objective Aspect of the Constituency

In tort law, judges have attempted to achieve a balance between a completely objective standard and a completely subjective standard by placing the reasonable person into the ‘position’ of the actor, or patient: as in the *Canterbury* test. The device of ‘position’ now allows, and obliges, the doctor to heed ‘particular incapacities’ of the actual patient (pg. 587) [8]. As explained by Osborne Reynolds:

The one big exception to the reasonable man’s unvarying personality and his use as a purely objective standard is that he normally assumes the physical characteristics of the actor, whether blind, crippled, or in excellent shape. These, it may be said, are among the circumstances in which the reasonable man is placed; or it may simply be admitted that in this regard we use a subjective standard (pg. 415) [19].

Hence, through the attention to ‘position’, neither the *Canterbury* test nor the ‘objective’ limb of the *Rogers* test is entirely objective; to the contrary, both involve a subjective element and, so, conceive ‘a partly “personalised” reasonable person’ (pg. 578) [8]. Thus, the ‘objective’ limb of the *Rogers* test of materiality, now adopted in *Montgomery*, is actually a composite of both objective and subjective elements.

The creation of a composite limb can be viewed as an attempt to find ‘a fit along a continuum of relevant characteristics rather than arguing for either extreme’ (pg. 84) [18]. Such a ‘fit’, or balance, would mean that some subjective characteristics of the actual patient can be allowed to outweigh the ‘reasonable man’s unvarying personality’ (pg. 415) [19]; although, other subjective characteristics could be trumped by objectivity, because extreme subjectivity is not permitted under the objective limb. In other words, the satisfaction an individual patient’s idiosyncrasies remain contingent, to some extent, upon the reasonable person’s objectivity. As such, in seeking to provide justification to a ‘reasonable person in the patient’s position’, the doctor has to attend to only some, but not all, of that patient’s idiosyncrasies. Now, this restriction on subjectivity provokes debate about the inclusion and exclusion of idiosyncrasies into the conception of a patient’s ‘position’; and, correspondingly, concern about excluded idiosyncrasies. The subjective limb of the *Rogers* test seeks to addresses this outstanding concern.

### The Subjective Aspect of the Constituency

The introduction of the subjective limb into the test of materiality would seem to be in response to concerns about excluding attention to any patient-idiosyncrasy, whatsoever it may be. The *Rogers* test, through its subjective limb, seeks to incorporate the extreme of subjectivity; although, the test limits extreme objectivity by coupling a reasonable patient with her ‘position’. As discussed above, ‘position’ is a point along the objective-subjective spectrum; and the ‘particular patient’ can be seen as a position at the subjective end of this spectrum. Hence, the distinction between ‘position’ and ‘particularity’ is blurred. As pointed out by Rob Heywood:

In terms of the actual need for a particular patient limb, it may well be that, in the majority of cases, there would be very little difference in outcome if, under the reasonable patient approach, greater emphasis was placed on what the reasonable person in the *patient’s position* would consider significant. Its specific inclusion, therefore, could be regarded as superfluous (pg. 460) [20].

But, if particularity is superfluous, then why would the Supreme Court, in *Montgomery*, have ‘refined’ Lord Scarman’s test? Heywood would seem to respond that the refinement was necessary ‘because all too often not enough emphasis was placed on the *patient’s position*, and so the circumstances of the patient were invariably overlooked’ (pg. 460) [20]. In making this response, Heywood relies on José Miola, who has pointed out that ‘position’ has been conceived as limited to the patient’s physical characteristics, to the exclusion of her social, cognitive, epistemic and emotional circumstances [21]; including her ‘particular fear or concerns’ (para 79) [17]. But, it is not clear *how* a doctor should apprehend a patient’s particular fears or concerns. In the Singaporean case of *Chok v Ooi* [22], which analyses *Montgomery*, Menon CJ makes it clear that:

The doctor has no open-ended duty to proactively elicit information from the patient, and will not be at risk of being found liable owing to the idiosyncratic concerns of the patient unless this was made known to the doctor or the doctor has reason to believe it to be so (para 145) [22].

It is equally clear that the doctor’s recognition of the patient’s particularity is not dependant on explicit questioning by the patient; rather, ‘There are a multitude of potential circumstances in which a court might find that the medical practitioner should have known of a particular fear or concern held by the patient’ (para 79) [17].

The ‘multitude of potential circumstances’ that should demonstrate a patient’s subjectivity to a doctor remain ambiguous. Nonetheless, regardless of questioning or any display of fears or concerns, the doctor is obliged to explain any ‘significant risk which would affect the judgment of a reasonable patient’ (para 65) [9]. Thus, the key to doctor’s duty, in seeking and obtaining a patient’s consent for medical treatment, is to first conceive a reasonable person: the recognition of, and response to, particularity flows fundamentally from the doctor’s conception of reasonability. This conception is discussed in the next section.

### The Reasonable Person: A Normative Ideal

There has been debate about the conception of a reasonable person. One proposition is that a reasonable person is a ‘positive’ conception which posits:

[T]he existence of a reasonable person who is not a real entity but a hypothetical construct…(that) can be approximated using empirically observable data. In other words, we can learn about the reasonable person by looking at the society. This implies that the reasonable person is in some sense a derivative of the society (pg. 371) [23].

In order to employ a positive conception in guiding consent practices, some form of public discussion or survey to seek the views of actual patients would be required [24]; as has been attempted in some health conditions [25]. Proponents of a positive conception argue that it is reflective of the behaviour or perspective of ordinary people, who ‘are marked by their common properties, like common intelligence and the fact that they do not all agree about what the true or correct life plan is’ (pg. 275) [26]. But, the fact of disagreement amongst ordinary persons also poses a strong objection to this conception; because, the idea of a reasonable person is to supply an objective standard that serves as a reference-point for all persons in a society and, therefore, it has to be agreed upon by all.

The countervailing proposition is that a reasonable person is a normative ‘ideal’ that is conceived by philosophical deliberation, and not by actual discussion amongst real people (pg. 371) [23]. By this conception, ‘reasonable’ refers to the theoretically-required, and not empirically-determined, characteristics of the people to whom decisions and actions have to be justified. In other words, the characterisation of a reasonable person deals with the elaboration of the features that this person *ought* to possess in order to qualify as the justificatory audience [23]. The implication is that some decisions about a particular issue can rightly be imposed on a diverse set of people, who have fundamental disagreements on that issue, if these decisions are justified by appeal to arguments that those persons, at some level of idealization, ought to accept on the basis of their membership of a society. This conception faces the objection that ‘an ideal conception would import the particular value system and form of good life held by those developing the ideal’ (pg. 275) [26]; and that it ‘introduces a standard that appeals not to an average person but a *better* person, such as those who are ideally careful and virtuous’ (pg. 81) [18].

In *Healthcare at Home Ltd v Common Services Agency* [27], Lord Reed JSC resolves the debate in favour of the conception of a reasonable person as a normative ideal. He explains that the reasonable person belongs ‘to an intellectual tradition of defining a legal standard by reference to a hypothetical person, which stretches back to the creation by Roman jurists of the figure of the *bonus paterfamilias’* (para 2) [27]. In English law, the reasonable person is often portrayed as the ‘man on the Clapman omnibus’ (para 1) [27]; who, Lord Reed JSC clarifies, is a ‘legal fiction’ and not an actual person (para 2) [27]. Lord Scarman had clarified similarly in *Sidaway*, in the specific context of consent for medical treatment, that a reasonable patient ‘is a norm (like the man on the Clapham omnibus), not a real person: and certainly not the patient himself’ (pg. 889A) [14]. Thus, Lord Scarman rejected a positive conception of a reasonable person; and Lord Reed JSC does likewise:

[I]t would (be) misconceived for a party to seek to lead evidence from actual passengers on the Clapham omnibus as to how they would have acted in a given situation or what they would have foreseen, in order to establish how the reasonable man would have acted in a given situation or what they would have foreseen. Even if the party offered to prove that his witnesses were reasonable men, the evidence would be beside the point. The behaviour of the reasonable man is not established by the evidence of witnesses, but by the application of a legal standard by the court. The court may require to be informed by evidence of circumstances which bear on its application of the standard of the reasonable man in any particular case; but it is then for the court to determine the outcome, in those circumstances, of applying that impersonal standard (para 3) [27].

The critical issue, then, is to imbue ‘that impersonal standard’ of the reasonable person with content; otherwise, the standard is hollow. As pointed out by Gardner, the practical requirement is for ‘substance to the reasonableness standard’ (pg. 288) [7]. Stated differently, what are the characteristics of this fictitious reasonable person, whom the doctor is required to place in the position of the patient; and, then, to whom the doctor should be able to justify treatment proposals that are made for the purpose of obtaining consent? Judges have not explicitly set out the characteristics of their ‘legal fiction’: these characteristics require to be inferred from their judgments. In order to do so, it is necessary to have a theoretical framework of reasonability so that judgments can be objectively analysed. This framework is discussed in the next section.

#### Which Normative Conception: Rawlsian or Feminist?

Apart from law, the reasonable person is a central idea in political philosophy. This idea features prominently in John Rawls’s widely-celebrated theory of justice [28]. Rawls deals with the problem of intractable disagreements amongst actual people in society by proposing the concept of ‘public reason’. He argues that fairness requires decision-makers to be able to supply reasons for their decisions and actions that would be acceptable to the ‘public’, in the form of a reasonable person. As per Rawls, being reasonable is ‘part of a political ideal of democratic citizenship that includes the idea of public reason’ (pg. 62) [28]. He proceeds to set out the characteristics of the ideal of a reasonable person—the ‘substance of the reasonableness standard’—which are discussed in the first sub-section below. The second sub-section points out the feminist objection to Rawls’s account, and it discusses an alternative conception of a reasonable person that emerges from the feminist ethic of care. These two conceptions of reasonability supply theoretical models that doctors could possibly use to imagine—to construct an ideal of—the reasonable patient; and these conceptions supply frameworks to analyse case law in order to identify the judicial conception of reasonability that doctors are required to follow.

### The Rawlsian Ideal of a Reasonable Person

Rawls commences his characterisation of a reasonable person with a discussion of rationality. His theory of justice assumes that all people are basically rational, and he explains that a rational person is one who can form and pursue her own views of a good life: she has the powers of deliberation and judgment to identify and assign priority to her own interests or ends, and to then choose effective means towards these ends (pg. 48) [28]. As such, a single-mindedly rational person would adopt means that are directed to the efficient attainment of her chosen end, without any other consideration. In contrast, according to Rawls, reasonable persons ‘are willing to govern their conduct by a principle from which they and others can reason in common; and reasonable people take into account the consequences of their actions on others’ well-being’ (pg. 49) [28].

Rawls conceives any person to have a rational aspect and a reasonable aspect to her personality: by her rational aspect, this person devises her own claims on society; and the move to reasonability then requires the person to assess the strength of her own claims by consideration to the claims of other members of society. Rawls discusses that the rational aspect of a person links to her idea of good, whereas the reasonable aspect relates to her idea of justice. In his words, ‘it is by the reasonable that we enter the pubic world of others and stand ready to propose, or to accept, as the case may be, fair terms of cooperation with them’ (pg. 53) [28]. Other philosophers, too, have made similar distinctions between rationality and reasonability [29].

Rawls discusses that the move from rationality to reasonability is characterised by two attributes: ‘reciprocity’ and acceptance of the ‘burdens of judgment’. He explains that reasonable persons display reciprocity by abiding to ‘fair terms of co-operation’ that are proposed to them, provided that they are ‘given the reassurance that others will likewise do so’ (pg. 136) [30]. As such, reciprocity may require a person to modify her rational ends, or to deviate from holding steadfastly to the most efficient means to achieve these ends, in order to ‘co-operate with others on terms all can accept’ (pg. 50) [28]. Rawls acknowledges that such co-operation may be beset with various disagreements and uncertainties, which he terms as the ‘burdens of judgment’ (pg. 56) [28]. Rawls describes these burdens of judgment as ‘the many hazards involved in the correct and conscientious exercise of our powers of reason and judgment in the ordinary course of political life’ (pg. 56) [28];^4^ and he argues that the ideal of reasonability requires the reasonable person to accept that proposals by others will be imbued with some such burdens of judgment. Thus, the Rawlsian conception of a reasonable patient would be a rational person who is willing to accept consent proposals that incorporate considerations of co-operation with other users of the NHS and acceptance of some uncertainties. The judicial approach to such a Rawlsian conception is discussed in the next section.

### The Reasonable Person as a care-recipient

Rawls’s work is set in the social contract tradition, with strongly liberal roots. Feminist scholars have criticized such liberal theories for being founded on ‘masculine’ conceptions of morality, with associated emphases on rights of individual choice and self-determination [31]. In contrast, feminists argue that people are embedded in society and cannot be abstracted from their social context and relations. An important premise, within the feminist argument, is that real people, as opposed to the ideal agent of theory, often simply do not possess the epistemic and cognitive abilities that would allow them to be rational in the first place. As such, the Rawlsian paradigm may fail, especially in the case of consent for medical treatment, because various apprehensions, biases and heuristics can lead to choices that do not reflect a patient’s actual preferences [32].

A seminal analysis of differences between masculine and feminist approaches to decision-making was set out by Carol Gilligan, who proposed the ‘ethic of care’ as an alternative to liberal theories of human interactions [33]. It seems apt to focus on care theory for the patient-doctor interaction; because, in its core guidance, *Good Medical Practice*, the GMC instructs that ‘Good doctors make the care of their patients their first concern’ (para 1) [34]. Therefore, it would seem that the GMC regards care as the guiding ethic for the medical profession, and it would follow that doctors should be mindful of this ethic whilst conceiving a reasonable patient.

Gilligan describes care as a ‘bond of attachment rather than a contract of agreement’ (pg. 57) [33]. This bond of attachment acknowledges power differences between the care provider (the doctor) and the care recipient (the patient), and it does not argue for a relationship between equals [35]. In the care paradigm, the reasonable patient would not expect the doctor to treat her as an equal and to involve her in all decisions; but, instead, to respect her through attention and responsiveness to her weaknesses and fallibilities. Furthermore, the care-oriented reasonable patient would not expect the doctor to always concede to her stated preferences; but, instead, to not abandon her to ill-judged choices [36]. The doctor would be expected to be cognizant that:

Attempts to care are continually challenged by a tension between expressed needs (those that arise within the one who needs) and inferred needs (those defined externally and imposed on the one said to have them). As carers, we cannot ignore expressed needs, but neither should we always indulge them (pg. 443) [37].

It is notable that care theories explicitly admit varying degrees of paternalism [35]. As argued by Nel Noddings, ‘despite classical liberalism’s fears of paternalism, a caring society must sometimes intervene in the lives of adults to prevent them from harming themselves’ (pg. 2) [36]. The judicial approach to a care-based conception of a reasonable patient is discussed in the next section.

**The judicial conception of a reasonable patient from*Montgomery***.

In *Montgomery*, Lord Kerr and Lord Reed JJSC observe that recent social and legal developments ‘point away from a model of the relationship between the doctor and the patient based on medical paternalism’ (para 81) [9]. Furthermore, their lordships consider that patients are ‘now widely regarded as persons holding rights, rather than passive recipients of care of the medical profession. They are also widely treated as consumers exercising choices’ (para 75) [9]. From these dicta, it would seem that a doctor should not conceive a reasonable patient as a care-recipient; and that paternalism, which is admitted (albeit sparingly) by care theorists, does not have any place in the patient-doctor relationship. Instead, as observed by Gardner [8], the judicial conception of a reasonable person seems to be set in a Rawlsian model, with implicit emphasis on a contractual relationship rather than the bond of attachment that is advanced by feminists. Although, as discussed below, the critically-nuanced Rawlsian approach to rationality is missing in case law.

As discussed earlier, Rawls’s reasonable person is characterised by the attributes of reciprocity and acceptance of the burdens of judgment. The *Montgomery* judgment is now explored for each of these attributes, in turn. Rawls’s idea of reciprocity as abiding to ‘fair terms of co-operation’ would suggest that, in addition to holding rights, reasonable patients have responsibilities, too. Such responsibilities might include recognising that the NHS has finite resources, and limiting demands on this service out of consideration for fellow-users of the NHS [38, 39]. If so, doctors could include such responsibilities in their conception of a reasonable patient. Prior to *Montgomery*, there are some instances of judicial reasoning along these lines. In *Airedale v Bland*, Sir Thomas Bingham MR acknowledged that:

An objective assessment of Mr Bland’s best interests, viewed through his eyes, would in my opinion give weight to…if altruism still lives, to a belief that finite resources are better devoted to enhancing life than simply averting death (pg. 813E) [40].

Not dissimilarly, in *R (on the application of McDonald) v Kensington and Chelsea* (a case about social care), Lady Hale JSC has conceded that ‘She (the patient) too can be expected to co-operate with the authority in choosing the most economical and acceptable way of meeting the need that she has’ (pg. 74A) [41]. But, *Montgomery* does not make any such concessions; and it would not seem to allow the doctor to conceive the consumer-patient as displaying reciprocity by accepting justification that was predicated on fair terms of co-operation with other patients.

Judges seem to have considered the second attribute of the Rawlsian reasonable person—acceptance of burdens of judgment—‘to be reduced to one of determining the extent of the knowledge which is to be attributed to the reasonable person standing in the position of the plaintiffs’ (pg. 686, Norse LJ) [42]. Traditionally, judges have allowed various assumptions about the knowledge that can be attributed to a reasonable person. For instance, a reasonable person would know, and not require to be warned of, the possibility of being hit by a cricket-ball when watching a match at Lord’s stadium, or being injured in a crash during a motor-racing show [43]. Similarly, in *Sidaway*, Lord Templeman reckoned that a reasonable person would fathom the ‘general danger of unavoidable and serious damage inherent in the operation’ from a description of the nature of the operation, and that the surgeon was ‘entitled to assume’ accordingly (pg. 903 A) [14]. But, in *Montgomery*, the Supreme Court does not seem to permit any such assumptions. At trial, it had been held that Mrs Montgomery was ‘clearly a highly intelligent person’ (para 246) [44], who could be assumed to be aware of the options of vaginal delivery and caesarean section and did not require to be informed of this choice. Nonetheless, the Supreme Court criticized the obstetrician for not informing Mrs Montgomery of the options. Implicitly, in the Supreme Court’s view, the obstetrician was not entitled to assume that any reasonable person would know of these options. Likewise, the GMC explicitly instructs doctors to ‘not rely on assumptions’ about the patient’s informational needs (para 12) [4].

Shorn of reciprocity and acceptance of the burdens of judgment, a reasonable person would be reduced to simply a rational person. Indeed, the Supreme Court’s emphasis on ‘informed choice’ and the patient’s entitlement ‘to be told of risks where that is necessary for her to make an informed decision’ (para 61) [9] points to a conception of a reasonable person as an entirely rational being. It has been pointed out that the model of informed decision-making is ingrained with propositions of the rational choice theory: that patients will have well-crystallised preferences and goals and, if they are supplied with material information, then they will be able to make treatment-choices that can attain these goals [45]. The premises can, in fact, be robustly challenged [46, 47]. Nonetheless, the law, as it emerges from *Montgomery*, would appear to instruct doctors to imagine a reasonable patient as a rational person; and, therefore, to make decisions that would be justifiable to entirely rational persons.

It could be claimed that the entirely rational reasonable-person that emerges from *Montgomery* represents an objective extreme that simply provides a starting point; which, then, requires to be modulated with subjectivity through the devices of ‘position’ and ‘particularity’. Indeed, the Supreme Court seems to have considered Mrs Montgomery’s short stature and diabetes, and associated anxieties about vaginal delivery, as her subjective features that indicated her preference for a caesarean section. However, in doing so, the Court maintained the traditional focus on ‘the physical characteristics of the actor’ (pg. 415) [19] as expressions of her subjectivity; and it did not consider her epistemic and cognitive characteristics, separately from her emotions. At trial, it had been established that Mrs Montgomery was a molecular biologist and a hospital-specialist for a pharmaceutical company (para 6), whose mother and sister were general medical practitioners (para 17) [44]. These characteristics would seem to be critically illuminative of Mrs Montgomery’s subjectivity, but do not seem to have influenced Supreme Court. Instead, the reference by Lord Kerr and Lord Reed JJSC to ‘No woman’ (para 94) [9] and by Lady Hale JSC to ‘any reasonable mother’ (para 113) [9] would suggest that they refrained from mitigating the objective demands of rationality by attention to subjectivity. This approach is surprising because Lord Kerr and Lord Reed JJSC caution explicitly that it ‘would therefore be a mistake to view patients as uninformed, incapable of understanding medical matters, or wholly dependent upon a flow of information from doctors’ (para 76) [9]; and it could be charged that the Supreme Court’s judgment is internally inconsistent.

Nonetheless, lower courts have followed the Supreme Court in disregarding epistemic and cognitive characteristics as illuminative of the patient’s subjectivity. For instance, in *Thefaut v Johnston* [48], it was pled that the surgeon was justified in making some assumptions about the patient’s awareness of the risks of an operation because the patient was an experienced midwife. Green J. dismissed this defence peremptorily:

A surgeon giving advice cannot quiz a patient about his or her state of knowledge and then trim down the advice accordingly. And nor can a surgeon simply make assumptions about an individual because it is known that the patient is professionally qualified. This would render the process arbitrary and subjective. It would also make the process needlessly over complicated since the far simpler course is to proffer full advice and not shape it according to the patient’s perceived state of knowledge. Assumptions of this sort should therefore not be made. The clinician should simply give the relevant complete advice’ (para 75) [48].

In contrast, in *Webster v Burton* [49], the Court of Appeal indicates that the patient’s ‘background (a university degree in nursing)’ (para 41) [49] should have been taken into consideration in obtaining her consent. However, the ‘background’ was not a concession that the doctor could imagine this patient to possess certain medical information. To the contrary, it was deemed to impose a requirement to provide additional information, over and above that to any reasonable person. Implicitly, the Court of Appeal considered that the patient’s epistemic status enhanced her rationality, rather than modulate it with reciprocity or acceptance of any burden of judgment. Fundamentally, reasonability hinged on rationality.

## Conclusion

Lord Reed JSC has observed that ‘The Clapham omnibus has many passengers. The most venerable is the reasonable man, who was born during the reign of Victoria but remains in vigorous health’ (para 1) [27]. His (or her) health apart, the reasonable person’s most overwhelming characteristic, from *Montgomery*, is his rationality. Accordingly, in dealing with the ‘buck’ of reasonability, doctors should conceive of patients as rational beings: people who will have clearly-defined health goals and abilities to choose treatments that are most likely to deliver these goals. In contemplating their decisions and actions, doctors should consider whether these can be justified to entirely rational people, who are not guided by any considerations other than the achievement of their own, well-crystallised ends.

Such a rational conception of a patient sits uneasily with duty of care that is advanced by the GMC in *Good Medical Practice* [34] and is widely regarded as the fundamental ethic for all healthcare professionals. As discussed earlier, care scholars recognise that real people are seldom entirely rational. The Supreme Court cites *Good Medical Practice* approvingly (para 77) [9], and it seems highly unlikely that the Court would have disregarded the obligations of care. Rather, as discussed by Emily Jackson, the Court may have viewed patients as ‘consumers’, and rejected their status as ‘recipients of care’, in a symbolic sense only, in order to emphasize the importance of providing patients with adequate information [47]. Indeed, it can be argued that the Supreme Court recognised the limitations of informed decision-making and, therefore, insisted on ‘dialogue’ (para 90) [9] between the patient and the doctor [46]. Nonetheless, doctors are left with little guidance about how to conceive what is ‘reasonable’ and what it not. In the metaphorical realm, doctors may now claim that the GMC and the law have not only ‘passed the buck’, but also sought, through the simultaneous and conflicting emphases on rationality and care, to ‘have their cake and eat it too’! Further work is urgently required in order to specify the doctor’s conception of a reasonable patient; otherwise, NHSR’s bill is likely to keep rising.

## Footnotes

1 Sometimes to a jury, but often back to the judges, themselves, when they occupy a dual role in civil cases.

2 Lord Scarman’s emphasis.

3 Although, in *Sidaway*, Lord Scarman was in the minority; the majority continued to endorse *Bolam.* So, technically, the justificatory constituency did not shift until the Supreme Court’s judgment in *Montgomery.*

4 By ‘political’, Rawls refers to basic social institutions upon which claims can be made by all citizens.

## Declarations

Yes.
